# A New Prenylated Flavanone from *Derris trifoliata* Lour

**DOI:** 10.3390/molecules17010657

**Published:** 2012-01-11

**Authors:** Cheng Jiang, Shengzi Liu, Weihong He, Xiongming Luo, Si Zhang, Zhihui Xiao, Ximin Qiu, Hao Yin

**Affiliations:** 1 Department of Pharmacy, Medical College, Hunan Normal University, Changsha 410006, China; Email: dangdangli@tom.com (C.J.); shengziliu@163.com (S.L.); 2 Key Laboratory of Marine Bio-resourses Sustainable Utilization, South China Sea Institute of Oceanology, Chinese Academy of Sciences, 164 West Xingang Road, Guangzhou 510301, China; Email: whhe@sisio.ac.cn (W.H.); luoxm163@sina.com (X.L.); zhsimd@scsio.ac.cn (S.Z.), xzh_77@yahoo.com.cn (Z.X.)

**Keywords:** *Derris trifoliate*, aerial part, prenylated flavanone, rotenoids, brine shrimp toxicity

## Abstract

A new flavanone, 4′,5,7-trihydroxy-6,8-di-(2-hydroxy-3-methylbut-3-enyl)- flavanone, was isolated from the aerial parts of *Derris trifoliate*, together with eleven known compounds: rotenone, tephrosin, 12a-hydroxyrotenone, deguelin, 6a,12a-dehydro-rotenone, dehydrodeguelin, 7a-*O*-methyldeguelol, 7a-*O*-methylelliptonol, 5,7,3',4'-tetra-hydroxy-6,8-diprenylisoflavone, daidzein and 4'-hydroxy-7-methoxyflavanone. 7a-*O*-Methylelliptonol was isolated for the first time from the genus *Derris*. Their structures were characterized on the basis of spectral data. Eight of the isolated compounds were found to be significantly toxic to brine shrimp (LC_50_ range 0.06–9.95 μg/mL). The new compound showed weak toxicity (LC_50_ = 211.31 μg/mL).

## 1. Introduction

*Derris trifoliata* Lour., a mangrove associate occurring in the tropical regions of Asia and East Africa [1], belongs to the Leguminosae, subfamily Papilionoideae. *D. trifoliata* is a poisonous plant, used locally for catching fish, and nowadays also used extensively for the control of insect pests [2]. Different parts of the plant were used in traditional medicine for treatment of wounds, calculus, rheumatism and dysmenorrhea and asthma [3]. Extracts and metabolites from this plant have been found to possess significant larvicidal, pesticidal, cytotoxic, anti-fungal, anti-inflammatory, antimicrobial, nitric oxide inhibitory, and cancer chemopreventive activities [4,5,6,7]. Previous phytochemical investigations on *D. trifoliata* show that flavonoids, including rotenoids, are the most characteristic compounds of this plant [8].

The brine shrimp lethality assay (BSLA) is considered a useful tool for assessment of toxicity, and has been widely used in chemical ecology studies on chemical defense in plants. The extract from aerial part of *D. trifoliata* was found to be toxic to brine shrimp (LC_50_ = 10.00 μg/mL) [9]. However, to the best of our knowledge, there are no reports on brine shrimp toxicity of compounds from *D. trifoliata*. As a part of our search for chemical defence compounds of mangrove plants in South China, a chemical investigation of the brine shrimp toxic extract from *D. trifoliata* has been carried out. In this study, we report the structural elucidation of a new prenylated flavanone, together with 11 known compounds from the aerial part of *D. trifoliata*, as well as their toxicity against brine shrimp.

## 2. Results and Discussion

**Figure 1:**
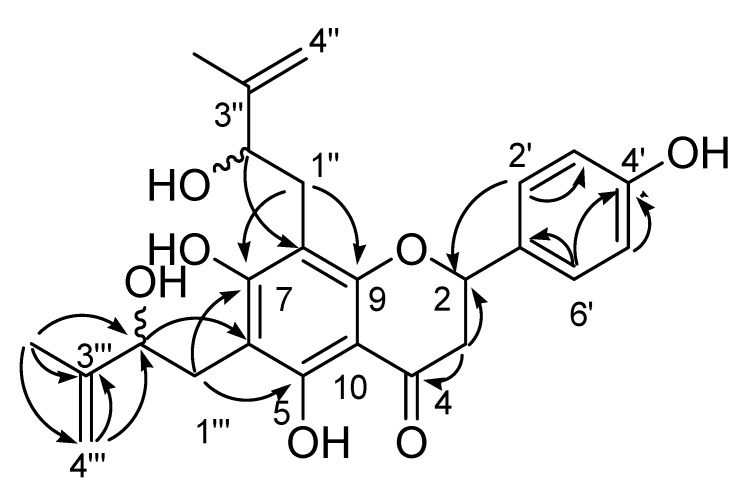
Structure of compound **1** and selected HMBC correlations.

Compound **1**, a light yellow, optically active oil ([α]_D_^20^ −17.3°, MeOH; c 0.3) oil, gave a molecular ion peak at *m/z* 440.1795 in the HREIMS, indicating a molecular formula of C_25_H_28_O_7_ (calc. 440.1835). The ^1^H-NMR spectrum of **1** showed three proton resonances at *δ*_H_ 2.76 (1H, m), 3.04 (1H, m), and 5.29 (1H, m), assignable to the typical H-2 and H-3 of a flavanone skeleton. The signals of H-3 overlapped with those of protons of other two methylenes (*δ*_H_ 2.82, 2H, m; and *δ*_H_ 2.95, 2H, m), but could be distinguished by HSQC spectra. In addition, resonances for an AA’XX’ spin system that is comprised of four protons (*δ*_H_ 6.82, 2H, d, *J* = 8.5 Hz, H-3′, 5′ and *δ*_H_ 7.25, 2H, d, *J* = 8.5 Hz, H-2′, 6′) assignable to a 4-subtituted B-ring were observed. Furthermore, together with HSQC spectrum, the 1D NMR (^13^C, ^1^H and DEPT) spectra of **1** revealed the presence of one ketone (*δ*_C_ 196.5, C-4), six aromatic carbons (*δ*_C_ 160.4, C-5; *δ*_C_ 106.5, C-6; *δ*_C_ 163.9, C-7; *δ*_C_ 105.2, C-8; *δ*_C_ 158.9, C-9; *δ*_C_ 102.6, C-10), two olefinic methylenes (*δ*_H_ 4.99, 1H, d, *J* = 7.5 Hz, H-4″a, *δ*_H_ 4.85, 1H, s, H-4″b, *δ*c 110.3, C-4″; *δ*_H_ 4.90, 1H, d, *J* = 7.5 Hz, H-4a, *δ*_H_ 4.78, 1H, s, H-4b, *δ*c 110.2, C-4), two quaternary olefinic carbons (*δ*c 146.8, C-3″; *δ*c 147.0, C-3), two methyls (*δ*_H_ 1.69, 3H, s, H-5″, *δ*c 18.4, C-5″; *δ*_H_ 1.83, 3H, s, H-5, *δ*c 18.3, C-5), two oxygenated methines (*δ*_H_ 4.28, 1H, t, *J* = 7.0 Hz, H-2, *δ*_c_ 77.0, C-2″; *δ*_H_ 4.28, 1H, t, *J* = 7.0 Hz, H-2″, *δ*c 77.0, C-2), and a hydroxyl (*δ*_H_ 12.41, 1H, s, OH-5). Comparison of the 1D NMR spectral data of compound **1** with those of 6-(2-hydroxy-3-methyl-3-butenyl)-8-prenyleriodictyol suggested that **1** was a flavnanone with two isoprenyls on ring A [10]. In the HMBC spectrum of **1**, the correlations from H-4 to C-2, 3, from H-5 to C-3, 4, and 2, and from H-1 to C-2, 3 confirmed the identity of the 2-hydroxy-3-methylbut-3-enyl moiety. The partial structure of another 2-hydroxy-3-methylbut-3-enyl in **1** was confirmed by HMBC correlation peaks of H-4″ with C-2″, 3″; H-5″ with C-3, 4; and H-1 with C-2, 3. Moreover, the substitution pattern of ring A was unambiguously established by HMBC correlations (Figure 1), which gave evidence for the placement of the two isoprenyls at C-6 and C-8, respectively. Thus, **1** was characterized as 4',5,7-trihydroxy-6,8-di-(2-hydroxy-3-methylbut-3-enyl)-flavanone. The absolute configuration of **1** was not determined.

Besides the new compound, 11 known compounds: Rotenone (**2**) [11], tephrosin (**3**) [12], 12a-hydroxyrotenone (**4**) [13], deguelin (**5**) [12], 6a,12a-dehydrorotenone (**6**) [11], dehydrodeguelin (**7**) [12], 7a-*O*-methyldeguelol (**8**) [14], 7a-*O*-methylelliptonol (**9**) [15], 5,7,3',4'-tetrahydroxy-6,8-diprenyl-isoflavone (**10**) [16], daidzein (**11**) [17], and 4'-hydroxy-7-methoxyflavanone (**12**) [18], were isolated from the plant and their structures elucidated by comparing their spectroscopic data with those reported in literature. As far as know, 7a-*O*-methylelliptonol is isolated for first time from the genus *Derris*.

Prenyl substitution is common among all known classes of flavonoids in *D. trifoliata*. However, the prenylation pattern in **1** is relatively rare. To the best of our knowledge, besides **1**, only two other flavonoids, 6,3'-di-(2-hydroxy-3-methylbut-3-enyl)-flavone, and 3,5'-di-(2-hydroxy-3-methylbut-3-enyl)-4,2',4'-trihydroxychalcone, were found to contain two 2-hydroxy-3-methylbut-3-enyls [19], and additionally compound **1** is the first flavonoid with two such groups on the same aromatic ring.

Results of the brine shrimp lethality assay on three crude extracts and nine isolated compounds are shown in Table 1. Eight flavonoids exhibited significant brine shrimp toxicity (LC_50_ < 30 μg/mL) [20]. The LC_50_ value obtained in present study for **2** was similar to those reported in literature: 0.049~1.8 μg/mL for rotenone [21,22]. The LC_50_ values for **3** and **4** reported here were somewhat lower than literature data, 2.2 μg/mL for tephrosin, and 1.9 μg/mL for 12a-hydroxyrotenone [10]. The brine shrimp toxicity of compounds **1**, **5–8**, and **12** had never been reported in literature. The rotenonoids with sp^3^ hybridizaiton at C-6a and C-12a, namely compounds **2–5**, showed extreme toxicity with LC_50_ values less than 1 μg/mL, and this is consistent with the previously reported cytotoxic effects of compounds from *D. trifoliata* [14]. 

**Table 1:** The mean LD_50_ values ± S.D for crude extracts and compounds screened against brine shrimp larvae (*Artemia salina*).

Extract/Compound	LC_50_ ± S.D. (μg/mL)
Petroleum ether extract	7.61 ± 1.62
Ethyl acetate extract	5.78 ± 1.18
*n*-Butanol extract	>500.00
Compound **1**	211.31 ± 12.5
Rotenone	0.06 ± 0.03
Tephrosin	0.24 ± 0.05
12a-Hydroxyrotenone	0.37 ± 0.15
Deguelin	0.73 ± 0.12
6a,12a-Dehydrorotenone	9.95 ± 1.05
Dehydrodeguelin	5.32 ± 0.6
7a-*O*-Methyldeguelol	2.75 ± 0.76
4'-Hydroxy-7-methoxyflavanone	3.97 ± 0.63

## 3. Experimental

### 3.1. General

Optical rotations were measured on a Jasco 1020 polarimeter. NMR spectra were obtained on a Bruker AVANCE 500 spectrometer (operating at 500 MHz for ^1^H-NMR, 125 MHz for ^13^C-NMR). HREIMS spectra were recorded on a Finnigan MAT TSQ 700 mass spectrometer. UV spectra were obtained in a Beckman DU-640 UV spectrophotometer. Semipreparative HPLC was carried out using a Phenomenex Luna 5 μL C18 100A ODS column (250 × 20 mm) on a system comprised of a Waters 600E Multisolvent Delivery System and a Waters 996 Photodiode Array Detector.

### 3.2. Plant Material

The aerial parts of *D. trifoliata* were collected in September 2008 from the mangrove area of Sanya, Hainan Province, Southern China. The material was identified by Prof. Si Zhang, Key Laboratory of Marine Bio-resourses Sustainable Utilization, South China Sea Institute of Oceanology, Chinese Academy of Sciences. A voucher specimen (No. GKLMMM22) is deposited at the herbarium of South China Sea Institute of Oceanology.

### 3.3. Extraction and Isolation

The dry powdered aerial part of *D. trifoliata* (7.0 kg) was extracted three times with ethanol-water (1:1, v/v, 40 L) at room temperature for 10 days. The pooled extracts were evaporated in *vacuo*. The resulting residue was suspended in H_2_O, and subsequently extracted with petroleum ether, ethyl acetate, and *n*-BuOH (7.5 L, room temperature, 10 h). After removal of solvent, the ethyl acetate extracts (65 g) was fractionated into 10 fractions (Fr-I~Fr-X) by column chromatography using a gradient of mixtures of CHCl_3_-MeOH (100:1 to 65:35). Fr-III (3.5 g) was subjected to a column chromatography eluting with a gradient of petroleum ether-ethyl acetate (5:1 to 2:1) to give four fractions (Fr-III-A~Fr-III-D). Fr-IV (2.5 g) was separated by column chromatography (petroleum ether-ethyl acetate (5:1 to 2:1 2.0 L)) to give four fractions (Fr-IV-A~Fr-IV-D). Fractions Fr-III-B (300mg), Fr-III-D (362 mg), and Fr-IV-A (400 mg) were further separated by semipreparative HPLC (liner gradient within 40 min from MeOH-H_2_O 7:3 to 10:0), respectively, to give **1** (7.0 mg; t_R_ = 14.5 min), **2** (14.0 mg; t_R_ 16.8 min), **3** (40.0 mg; t_R_ 18.1 min), **4** (20.0 mg; t_R_ 16.4 min), **5** (3.5 mg; t_R_ 17.9 min), **6 **(2.5 mg; t_R_ 24.4 min), **7** (2.5 mg; t_R_ 26.4 min), **8** (4.0 mg; t_R_ 10.9 min), and **9** (5.0 mg; t_R_ 21.4 min). Fr-V (5.3 g) was separated by column chromatography over a silica gel using gradient elution of CHCl_3_-ethyl acetate (9:1 to 7:3) to give four fractions (Fr-V-A~Fr-V-D). Fr-V-A (250 mg) and Fr-V-B (120 mg) were further purified by semipreparative HPLC with liner gradient within 40 min from MeOH-H_2_O from 1:1 to 1:0 to afford **10** (2.5 mg; t_R_ 32.6 min). **11** (4.0 mg; t_R_ 8.7 min) and **12** (4.3 mg; t_R_ 13.7 min).

*4’,5,7-Trihydroxy-6,8-di-(2-hydroxy-3-methylbut-3-enyl)-flavanone* (**1**): A light yellow oil, [α]_D_^20^ −17.3° (MeOH; c 0.3), HREIMS *m/z*: 440.1795 [M]^+^; UV (MeOH) *λ*_max_ nm: 298.3, 345.9; ^1^H-NMR (CDCl_3_): *δ*_H_ = 5.29 (1H, m, H-2), 2.76 (1H, m, H-3a), 3.04 (1H, m, H-3b), 6.82 (2H, d, *J* = 8.5 Hz, H-3′, 5′), 7.25 (2H, d, *J* = 8.5 Hz, H-2′, 6′), 2.82 (1H, m, H-1″a), 3.08 (1H, m, H-1″b), 4.31 (1H, t, *J* = 7.0 Hz, H-2″), 4.99 (1H, d, *J* = 7.5 Hz, H-4″a), 4.85 (1H, s, H-4″b), 1.69 (3H, s, H-5″), 2.82 (1H, m, H-1a), 3.08 (1H, m, H-1b), 4.28 (1H, t, *J* = 7.0 Hz, H-2), 4.90 (1H, d, *J* = 7.5 Hz, H-4a), 4.78 (1H, s, H-4b), 1.83 (3H, s, H-5″), 12.41 (1H, s, OH-5); ^13^C-NMR (CDCl_3_): *δ*_c_ = 78.8 (C-2), 43.0 (C-3), 196.5 (C-4), 160.4 (C-5), 106.5 (C-6), 163.9 (C-7), 105.2 (C-8), 158.9 (C-9), 102.6 (C-10), 130.5 (C-1′) 127.7 (C-2′,6′), 115.6 (C-3′,5′), 156.3 (C-4′), 29.4 (C-1″), 77.0 (C-2″, C-2, interchangeable with C-2), 146.8 (C-3″), 110.3 (C-4″), 18.4 (C-5″), 28.6 (C-1), 77.0 (C-2, interchangeable with C-2″), 147.0 (C-3), 110.2( C-4), 18.3 (C-5).

### 3.4. Brine Shrimp Assay

Toxicity against brine shrimp was evaluated using the method reported by Wanyoike [20]. Artificial seawater was prepared by dissolving sea salt (38 g) in distilled water (1 L). The 60-mm culture dishe were used for hatching. To seawater (50 mL), brine shrimp eggs (10 mg) were added. The culture dish was placed in the light incubator at 25 °C for 48 h. The larvae were attracted to one side of the vessel with a light source and collected with pipette. Nauplii were separated from eggs by aliquoting them three times in small beakers containing seawater. The brine shrimp are then put in wells of a 96 flat-bottom well plate (Nunc Microwell plate). Each column (12 per plate) contains a two-fold dilution series representing 8 doses. Potassium dichromate was used as a positive control, as well as the solvent DMSO was used as a negative control. Each sample was repeated six times (columns) in each experiment. After 24 hours of exposure to the samples, the number of died and total (determined after killing by freezing) brine shrimp for each well were determined. The data were analyzed by probit regression (SPSS) to calculate the LC_50_.

## 4. Conclusions

A new prenylated flavanone **1** was isolated from aerial parts of *D. trifoliata*, and its structure was elucidated on the basis of spectroscopic analysis as 4′,5,7-trihydroxy-6,8-di-(2-hydroxy-3-methylbut-3-enyl)-flavanone. Among the isolated compounds, all rotenoids **2–9**, and a flavanone **12** exhibited significant toxicity against brine shrimp, while the new flavanone **1** showed low brine shrimp lethality.

## Supplementary Materials

Supplementary materials can be accessed at: http://www.mdpi.com/1420-3049/17/1/657/s1.

## References

[1] Shah D.G., Bhatt S. (2008). First Record of the Mangrove Associate Derris Trifoliata Lour. from Gujarat. JBNHS.

[2] Witt J.D., Warren S.L., Ranney T.G., Baker J.R. (1999). Biorational and Conventional Plant Protectants Reduce Feeding by Adult Japanese Beetles. J. Environ. Hortic..

[3] Kirtikar K.R., Basu B.D. (1987). Indian Medicinal Plants.

[4] Bhattacharyya A., Babu C.R. (2009). Purification and Biochemical Characterization of a Serine Proteinase Inhibitor from Derris trifoliata Lour. Seeds: Insight into Structural and Antimalarial Features. Phytochemistry.

[5] Khan M.R., Omoloso A.D., Barewai Y. (2006). Antimicrobial Activity of the Derris Elliptica, Derris Indica And Derris Trifoliata Extractives. Fitoterapia.

[6] Tewtrakul S., Cheenpracha S., Karalai C. (2009). Nitric Oxide Inhibitory Principles from Derris trifoliata Stems. Phytomedicine.

[7] Yenesew A., Twinomuhwezi H., Kabaru J.M., Akala H.M., Kiremire B.T., Heydenreich M., Peter M.G., Eyase F.L., Waters N.C., Walsh D.S. (2009). Antiplasmodial and Larvicidal Flavonoids from Derris trifoliata. Bull. Chem. Soc. Ethiop..

[8] Gomes C.M.R., Gottlieb O.R., Bettolo G.B.M., Dellemonache F., Polhill R.M. (1981). Systematic Significance of Flavonoids in Derris and Lonchocarpus. Biochem. Syst. Ecol..

[9] Mamoon S.A., Azam M.G. (2011). Diuretic Activity and Brine Shrimp Toxicity of Derris trifoliata Lour. IJPLS.

[10] Seo E.K., Silva G.L., Chai H.B., Chagwedera T.E., Farnsworth N.R., Cordell G.A., Pezzuto J.M., Kinghorn A.D. (1997). Cytotoxic Prenylated Flavanones from Monotes engleri. Phytochemistry.

[11] Carlson D.G., Weislede D., Tallent W.H. (1973). Nmr Investigations of Rotenoids. Tetrahedron.

[12] Andrei C.C., Viera P.C., Fernandes J.B., daSilva M.F.D.F., Fo E.R. (1997). Dimethylchromene Rotenoids from *Tephrosia candida*. Phytochemistry.

[13] Magalhaes A.F., Tozzi A.M.G.A., Sales B.H.L.N., Magalhaes E.G. (1996). Twenty-three Flavonoids from *Lonchocarpus subglaucescens*. Phytochemistry.

[14] Chantrapromma S., Fun H.K., Pullaput Y., Wongtap H., Dejmanee S., Chantrapromma K. (2005). 7a-*O*-methyldeguelol. Acta Crystallogr. Sect. E Struct. Rep. Online.

[15] Cheenpracha S., Karalai C., Ponglimanont C., Chantrapromma K. (2007). Cytotoxic rotenoloids from the stems of *Derris trifoliata*. Can. J. Chem..

[16] Singhal A.K., Sharma R.P., Thyagarajan G., Herz W., Govindan S.V. (1980). New Prenylated Isoflavones and a Prenylated Dihydroflavonol from *Millettia pachycarpa*. Phytochemistry.

[17] Lu H.-Y., Liang J.-Y., Yu P., Qu W., Zhao L. (2008). Isoflavones and their Derivatives from the Root of Derris elliptica (Roxb.) Benth. Chin. J. Nat. Med..

[18] Camarda L., Merlini L., Nasini G. (1977). Novel 4-styrylflavans from Gum Accroides. Can. J. Chem..

[19] Barron D., Ibrahim R. (1996). Isoprenylated Flavonoids-a Survey. Phytochemistry.

[20] Wanyoike G.N., Chhabra S.C., Lang’at-Thoruwa C.C., Omar S.A. (2004). Brine Shrimp Toxicity and Antiplasmodial Activity of Five Kenyan Medicinal Plants. J. Ethnopharmacol..

[21] Colman-Saizarbitoria T., Montilla L., Rodriguez M., Castillo A., Hasegawa M. (2009). Xymarginatin: A New Acetogenin Inhibitor of Mitochondrial Electron Transport From Xylopia Emarginata Mart., Annonaceae. Rev. Bras. Farmacogn..

[22] Alali F., Zeng L., Zhang Y., Ye Q., Craig Hopp D., Schwedler J.T., McLaughlin J.L. (1997). 4-deoxyannomontacin and (2,4-cis and trans)-annomontacinone, new bioactive mono-tetrahydrofuran annonaceous acetogenins from Goniothalamus giganteus. Bioorg. Med. Chem..

