# Supplementary figures and images for: A New Prenylated Flavanone from Derris trifoliata Lour
Source: Molecules. 2012 Jan 11;17(1):657–63. doi: 10.3390/molecules17010657 (PMC6268681; doi:10.3390/molecules17010657)

# <sup>1</sup>H NMR Spectrum of JC-2011-11

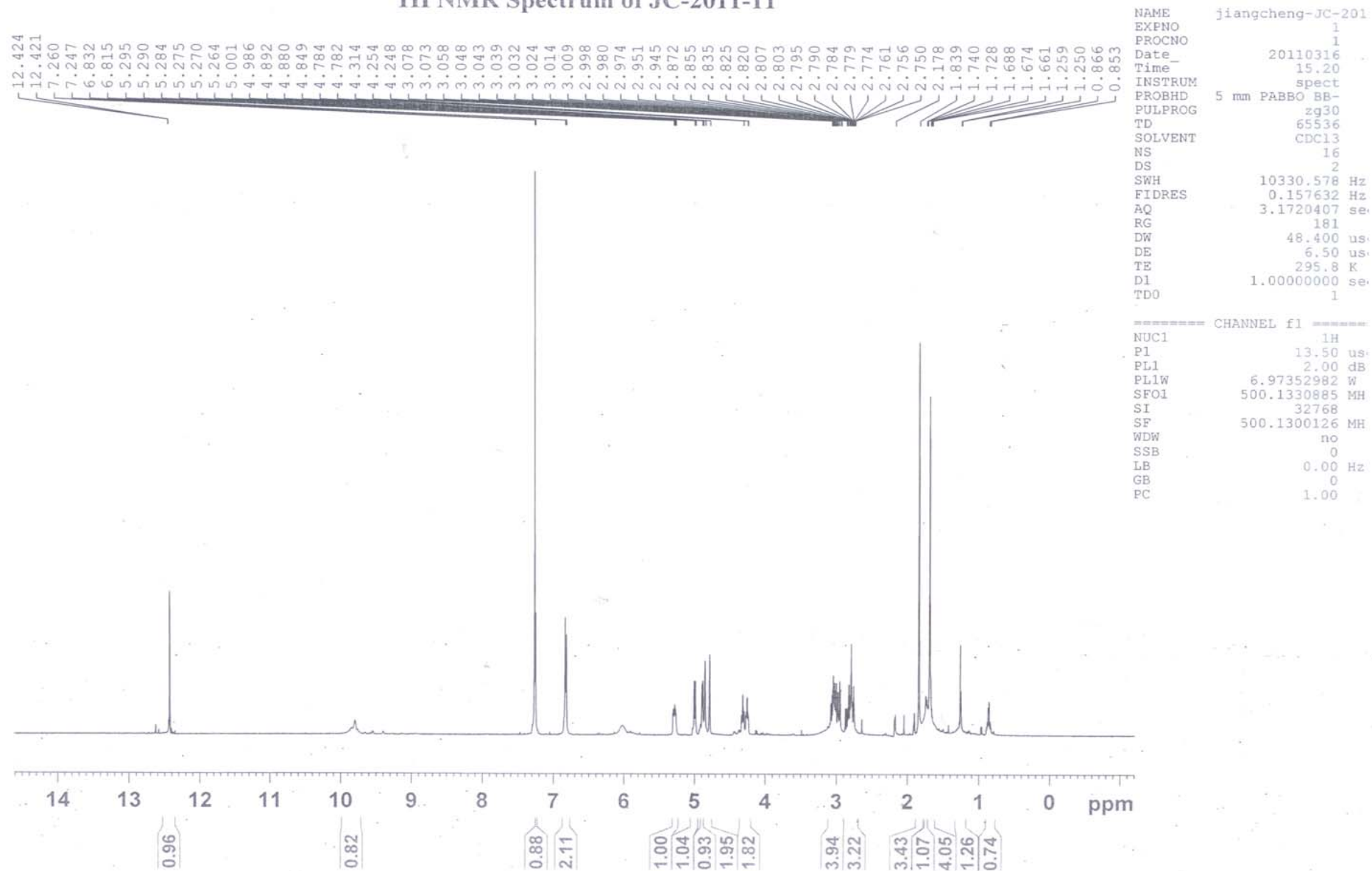

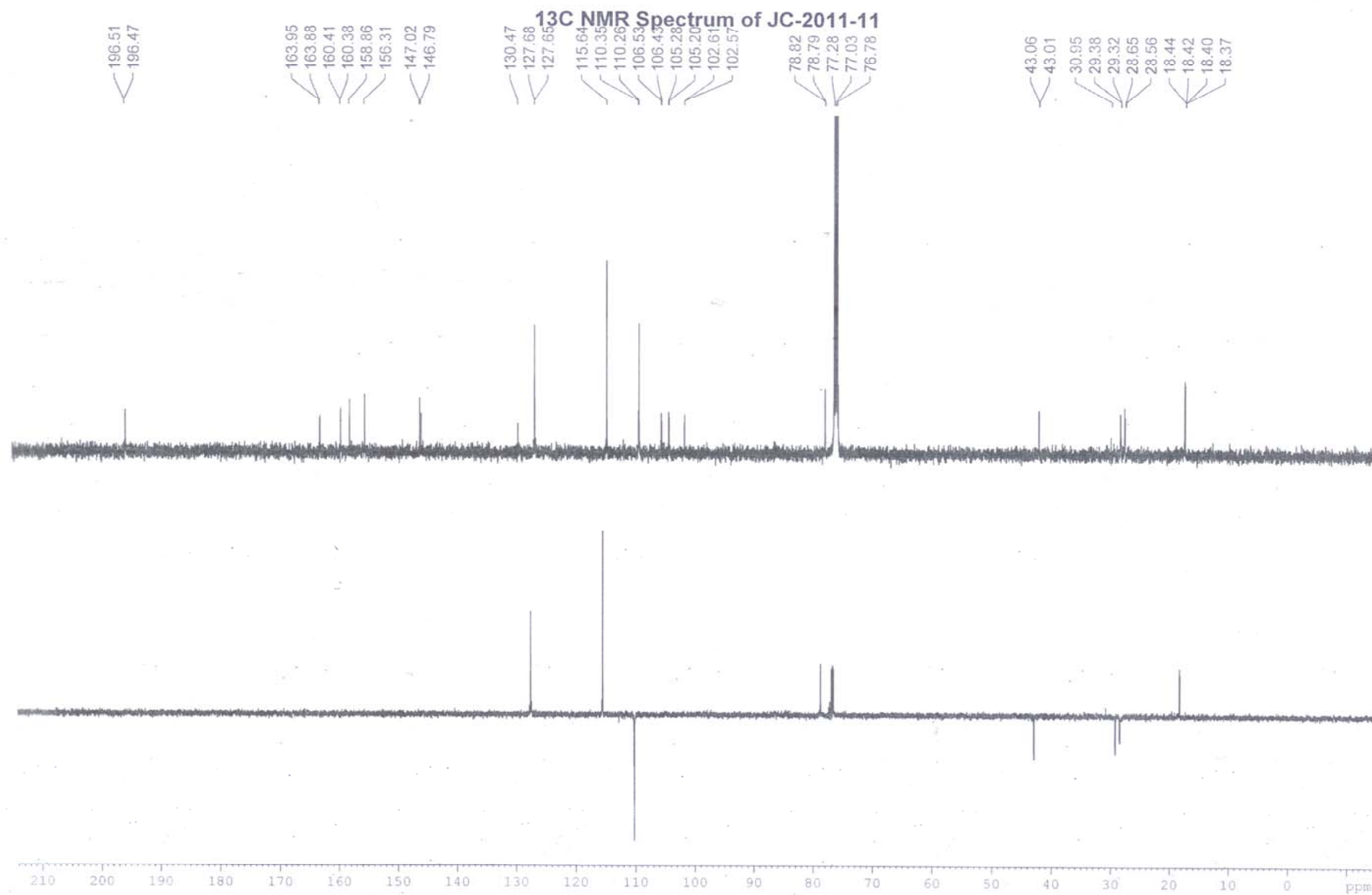

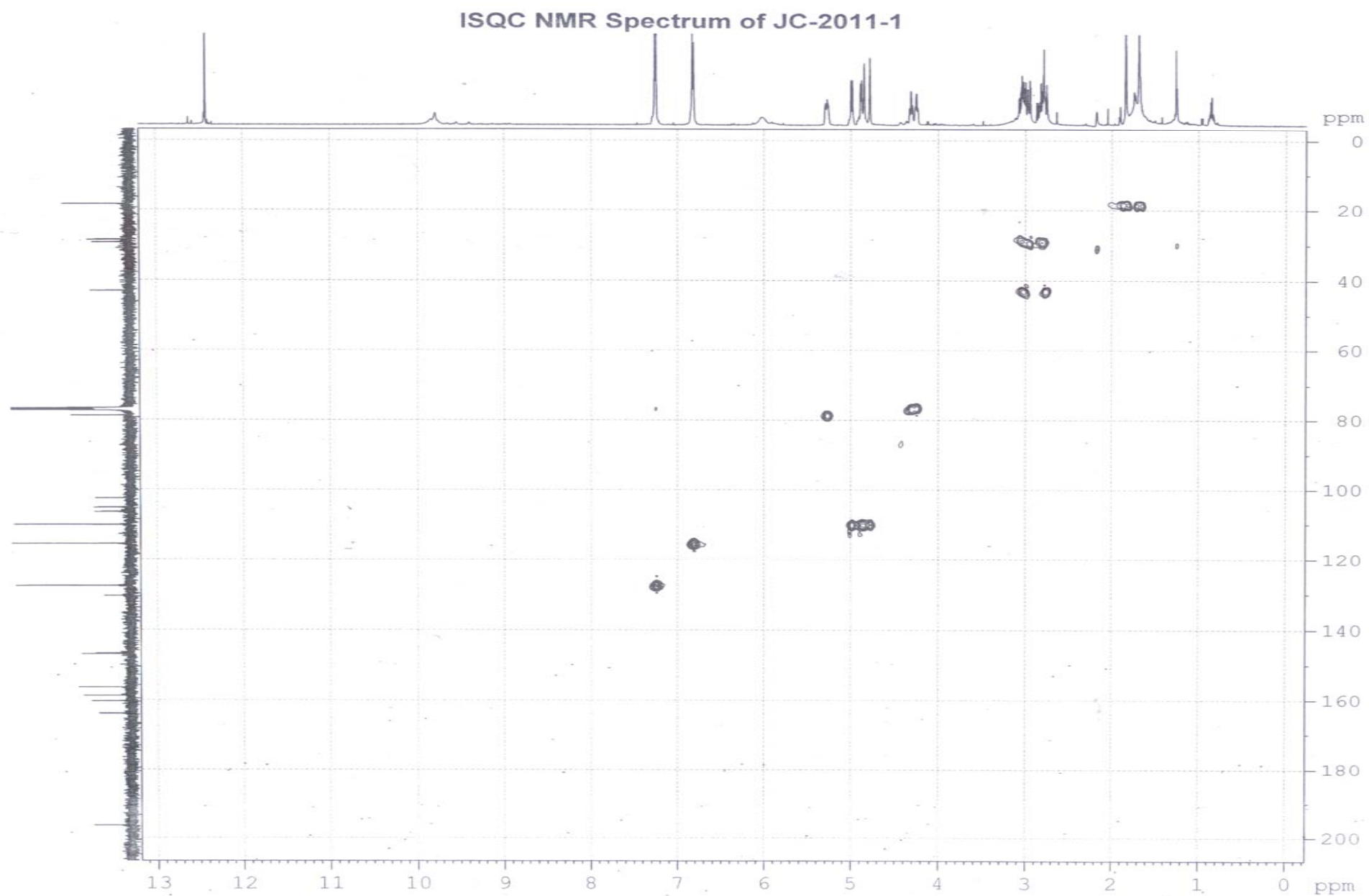

## HMBC NMR Spectrum of JC-2011-11

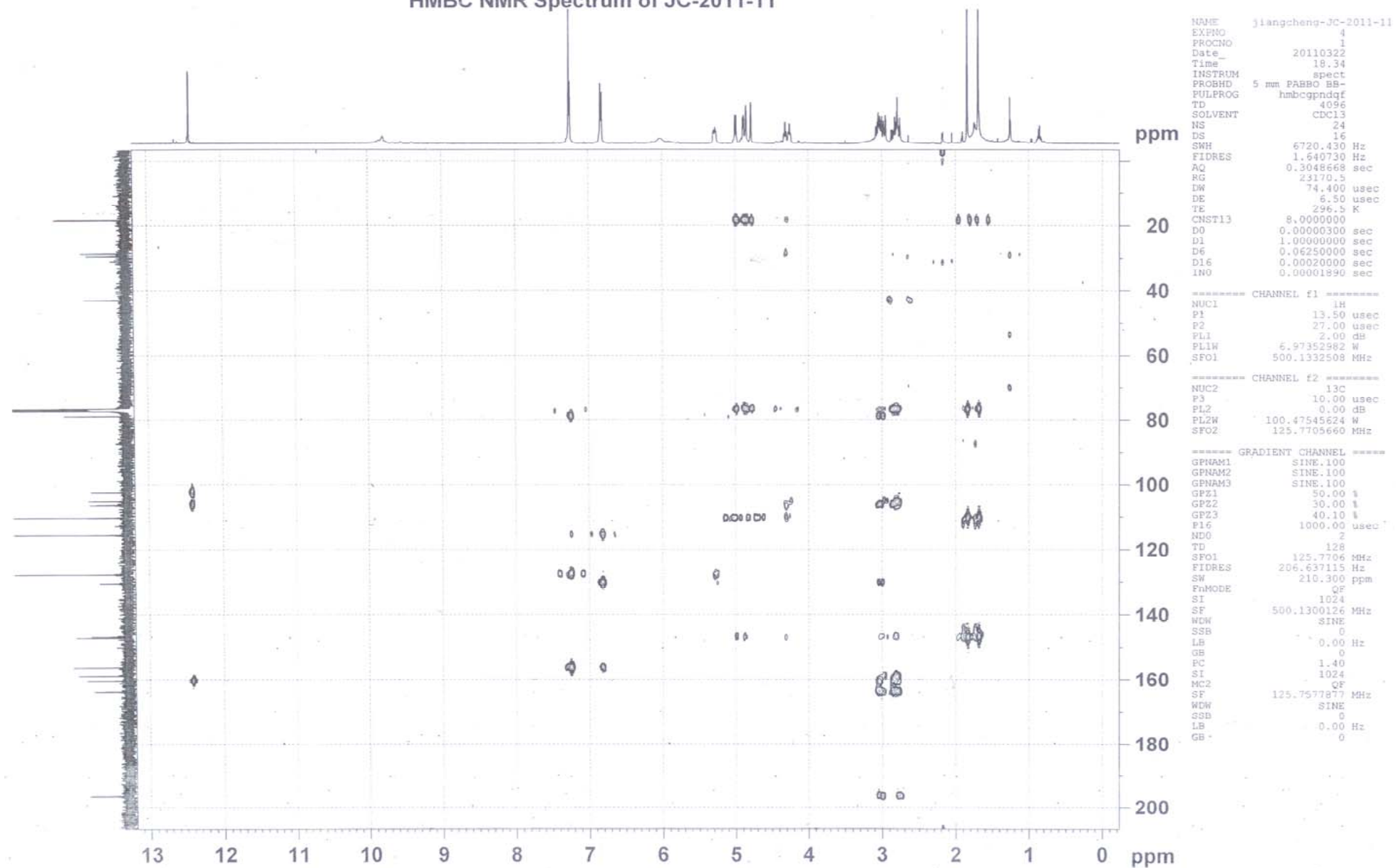

Supplement: Supplementary file 1 [file molecules-17-00657-s001.pdf]
